# Vaccination of Dr. Amthor’s Sons

**Published:** 1897-08

**Authors:** Montague R. Leverson

**Affiliations:** Fort Hamilton, L. I., N. Y.


### VACCINATION OF DR. AMTHOR'S SONS.

Fort Hamilton, L. I., N. Y., July 12th, 1897.

Editor of The Homœopathic Physician.

On page 205 of your June number you publish a letter from Elias C. Price, of Baltimore, announcing the death of Dr. J. M. R. Amthor. In concluding his letter he states that three of Dr. Amthor's sons died of small-pox, "he having neglected to vaccinate them."

In answer I will state that for every case of a person dying of small-pox who was *un*vaccinated I can produce at least three who have died of the same disease who *had* been vaccinated.

I invite Dr. Elias C. Price to answer my pathological table of small-pox, cow-pox, and syphilis. This shows conclusively that cow-pox and syphilis are the same disease and both distinct from small-pox.

If he will read the abstract of the testimony before the Royal British Commission now publishing in your journal, although thus far only the evidence of pro-vaccinist witnesses has been given, he will see that even that testimony demonstrates the uselessness of this "grotesque superstition."

Respectfully,

MONTAGUE R. LEVERSON.

